# Estrogenic Effect of Various Plant Extracts on Eel (*Anguilla japonica*) Hepatocytes

**DOI:** 10.3390/molecules30132781

**Published:** 2025-06-27

**Authors:** Jeong Hee Yoon, Ji Eun Ha, Joon Yeong Kwon

**Affiliations:** Department of Aquatic Life and Medical Science, Sunmoon University, Asan 31460, Republic of Korea; jeonghee3937@naver.com (J.H.Y.); nancy99ha@naver.com (J.E.H.)

**Keywords:** phytoestrogens, *Anguilla japonica*, hepatocytes, vitellogenin

## Abstract

Estrogen plays some important roles in many physiological processes in animals. This hormone is used as a type of medication for humans and animals, including fish, but is associated with serious side effects and environmental persistence, which has led to a growing interest in phytoestrogens as an alternative. Phytoestrogens are compounds derived from plants that are structurally similar to estrogen and may exhibit similar behavior in the body. To date, no studies have investigated the activity of phytoestrogens in relation to the maturation of eels. In the present study, we investigated the effects of ten different plant extracts on vitellogenin (*vtg*) and estrogen receptor (*esr1*, *esr2*) gene expression in eel hepatocytes. As a result, Schisandra and Astragalus extracts induced higher levels of *vtg* mRNA expression compared to the other extracts. However, increased *esr* mRNA expression was observed only in the Schisandra and soybean extract-treated groups. The phytoestrogens known to be present in Schisandra and Astragalus were analyzed using HPLC. Schizandrin, gomisin A, and gomisin N were detected in Schisandra extract, and calycosin and formononetin were detected in Astragalus extract. We then examined whether these phytoestrogens could induce *vtg* mRNA expression in eel hepatocytes. As a result, gomisin N and formononetin significantly induced *vtg* mRNA expression. In conclusion, among the 10 plant extracts treated in this study, Schisandra and Astragalus extracts induced estrogenic activity in eel hepatocytes. These extracts were found to contain phytoestrogens, with gomisin N and formononetin identified as the primary active components responsible for the observed estrogenic activity in eel hepatocytes.

## 1. Introduction

Estrogen is involved in regulating reproductive cycles and maintaining the function of the skeletal and cardiovascular systems in mammals. Estrogens include three major forms, estrone (E1), 17β-estradiol (E2), and estriol (E3), which act by binding to estrogen receptors (esr1 and esr2) in target tissues. Deficiency of this hormone can increase the risk of various diseases [1,2,3] and may require treatment with estrogen-based medicines [4,5]. Common estrogen-based medicines include mestranol, ethinylestradiol, and estradiol valerate.

Estrogens are also implicated in fish reproduction, including in eels (*Anguilla japonica*), which are a major aquaculture species of high economic value in East Asia. Due to their complex life history, eels do not naturally undergo sexual maturation in captivity. Therefore, artificial induction of maturation through repeated administration of hormones is being actively studied. In European eels, one type of estrogen, E2, in combination with other steroids was capable of inducing vitellogenesis more effectively than conventional pituitary extracts, implying the possible use of E2 as a key player, as shown in a previous study [6]. However, using exogenous estrogens for mammals and fish is not always safe because they can cause adverse physiological effects and persist in the environment [7,8].

There has been an increasing interest in phytoestrogens, which can replace the physiological effects of estrogen [9]. Phytoestrogens are plant-derived compounds that are broadly categorized into flavonoids (isoflavones and coumestans) and non-flavonoids (lignans) [10,11]. They are found in various plants such as soy, clover, alfalfa, and flaxseed [12]. Phytoestrogens have a phenolic A ring that is structurally similar to estrogen [13]. They can bind to the estrogen receptor (esr) in the body and exhibit estrogen-like physiological effects [9].

Few studies so far have investigated the estrogenic effects of various plant extracts on eel reproduction. In this study, we sought to discover phytoestrogen sources that could be applicable to eels as alternatives to E2. Ten plants known to contain phytoestrogens were selected based on their purchasability and price. These plants are Schisandra (schisandrin), Astragalus (formononetin), guava (quercetin), alfalfa (coumestrol), onion (quercetin), turmeric (curcumin), garlic (myricetin), kudzu (puerarin), soybean (genistein), and flaxseed (secoisolariciresinol), all of which are known to contain bioactive compounds representative of their respective phytoestrogen categories [14,15,16,17,18,19,20,21]. Many of these plants have also been traditionally used in herbal medicine and have been reported to exhibit hormone-like or reproductive health-related pharmacological effects in mammals [22,23,24,25,26,27].

In the process of endocrine regulation in fish, gonadotropic hormones secreted by the pituitary gland stimulate the gonads to secrete estrogen. Hepatocytes synthesize and secrete vitellogenin (vtg) in response to esr-bound estrogen [28,29]. Eel hepatocytes have also been reported to induce vtg in response to estrogen [30], making them suitable for evaluating the estrogenic activity of plant extracts in vitro.

This study aimed to evaluate and compare the estrogenic activity of ten phytoestrogen-rich plant extracts on eel hepatocytes and identify specific active compounds responsible for their effects.

## 2. Results

### 2.1. Analysis of Hepatocyte Viability After Treatment with Plant Extracts

**Figure 1:**
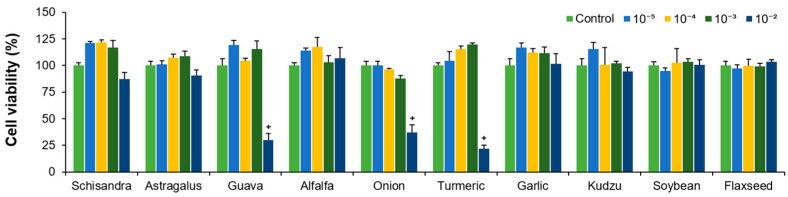
Cell viability of eel hepatocytes after treatment with extracts from Schisandra, Astragalus, guava, alfalfa, onion, turmeric, garlic, kudzu, soybean, and flaxseed. Extracts were applied at serially diluted concentrations (10^−^^5^ to 10^−^^2^ (*v*/*v*)) from the undiluted stock solutions. Error bars represent ± the standard error of the mean. + indicates significantly lower than the control (*p* < 0.05).

Eel hepatocytes were treated with ten different plant extracts at various concentrations. Most of the plant extracts had no significant effect on cell viability at concentrations below 10^−3^ (*v*/*v*). However, at a concentrations of 10^−2^ (*v*/*v*), viability was significantly reduced in guava, onion, and turmeric extract treatments (Figure 1, *p* < 0.05). Therefore, in subsequent experiments, concentrations of 10^−5^ to 10^−3^ (*v*/*v*) were used, which did not affect cell viability.

### 2.2. Analysis of vtg, esr1, and esr2 mRNA Expression in Hepatocytes After Plant Extract Treatments

**Figure 2:**
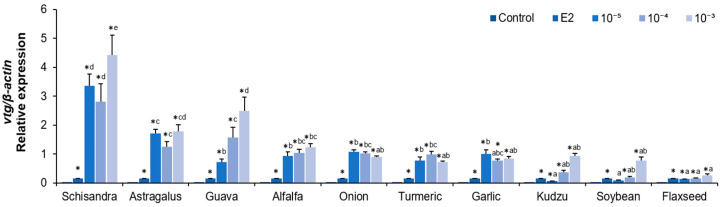
Relative expression levels of *vtg* mRNA normalized to *β-actin* in eel hepatocytes after treatment with plant extracts from Schisandra, Astragalus, guava, alfalfa, onion, turmeric, garlic, kudzu, soybean and flaxseed. Extracts were applied at serial dilutions of 10^−^^5^, 10^−^^4^, and 10^−^^3^ (*v*/*v*) from stock solutions. * indicates significantly higher than the control (*p* < 0.05). Different alphabetical superscripts indicate significant differences among extract treatments (*p* < 0.05).

After the treatment of hepatocytes with 10 plant extracts, the expression of *vtg* mRNA was significantly increased in most extracts except for the 10^−5^ (*v*/*v*) soybean extract compared to the control (Figure 2, *p* < 0.05). In particular, the highest *vtg* mRNA expression was observed in the 10^−3^ (*v*/*v*) treatment of the Schisandra, Astragalus, and guava extracts, with the Schisandra and Astragalus extracts showing consistently high expression at all concentrations (Figure 2, *p* < 0.05).

**Figure 3:**
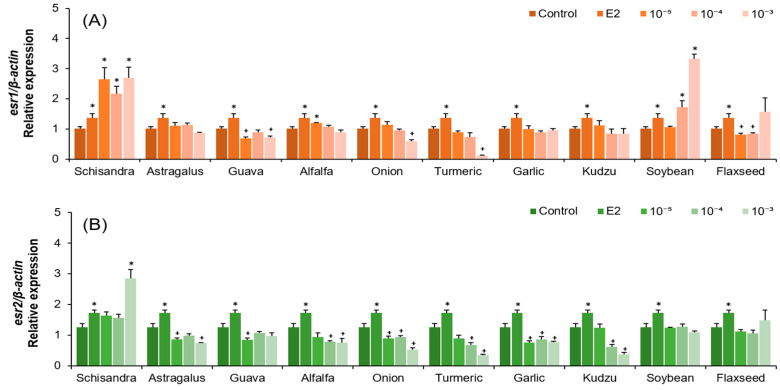
(**A**) Relative expression of *esr1* mRNA and (**B**) relative expression of *esr2* mRNA normalized to *β-actin* in eel hepatocytes after treatment with plant extracts from the indicated sources. Extracts were applied at 10^−^^5^, 10^−^^4^, and 10^−^^3^ (*v*/*v*) dilutions from stock solutions. * indicates significantly higher than the control (*p* < 0.05). + indicates significantly lower than the control (*p* < 0.05).

For *esr1* mRNA, its expression was significantly increased in the Schisandra extract treatment compared to the control at all concentrations, and increases were also seen at some concentrations of alfalfa and soybean extracts (Figure 3A, *p* < 0.05). On the other hand, guava, onion, turmeric, and flaxseed extracts significantly decreased the expression (Figure 3A, *p* < 0.05), while Astragalus, garlic, and kudzu extracts did not show significant differences.

The expression of *esr2* mRNA remained unchanged or tended to decrease significantly in most plant extracts, with a significant increase only at the 10^−3^ (*v*/*v*) concentration of the Schisandra extract (Figure 3B, *p* < 0.05). Soybean and flaxseed extracts did not show any difference at any concentration.

### 2.3. HPLC (High-Performance Liquid Chromatography) Analysis of Phytoestrogens in Schisandra and Astragalus Extracts

**Figure 4:**
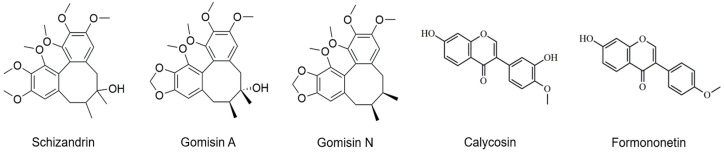
Chemical structures of phytoestrogen standards used in this study: schizandrin, gomisin A, gomisin N, calycosin, and formononetin.

**Figure 5:**
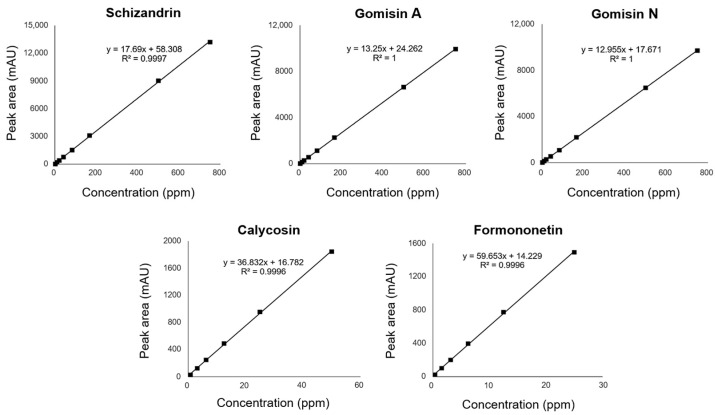
Calibration curves of standard compounds (schizandrin, gomisin A, gomisin N, calycosin, and formononetin) used for HPLC analysis. The ranges of tested concentration were as follows: calycosin, 0.78–50 ppm; formononetin, 0.39–25 ppm; and schizandrin, gomisin A, and gomisin N, 2.08–750 ppm. Each calibration curve showed excellent linearity (R^2^ > 0.999).

**Figure 6:**
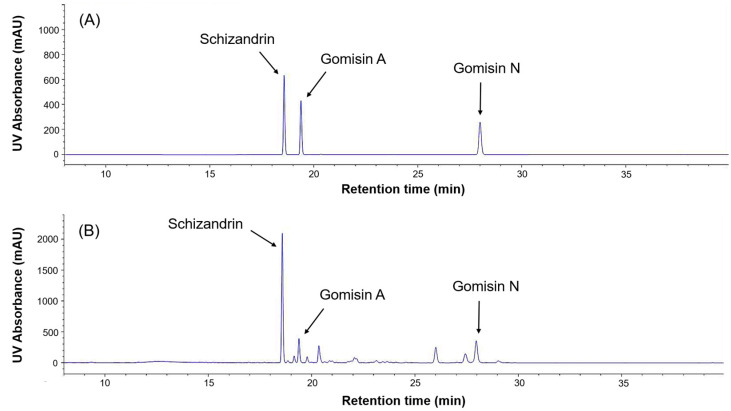
HPLC analysis for the measurement of phytoestrogens (schizandrin, gomisin A, gomisin N) in the Schisandra extract. (**A**) HPLC-DAD chromatogram of standard compounds (schizandrin, gomisin A, and gomisin N). (**B**) HPLC-DAD chromatogram of the Schisandra extract.

**Figure 7:**
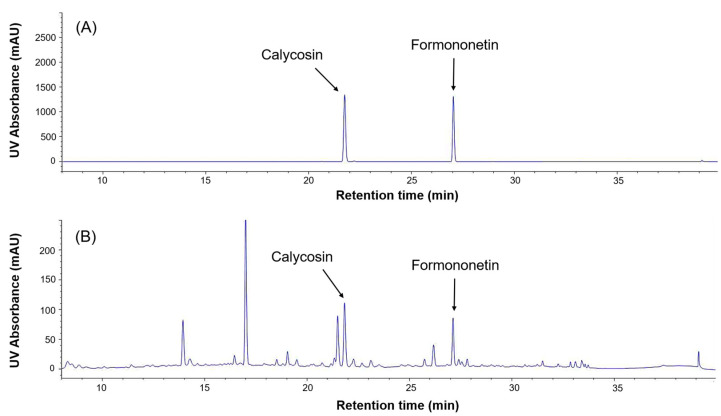
HPLC analysis for the measurement of phytoestrogens (calycosin and formononetin) in the Astragalus extract. (**A**) HPLC-DAD chromatogram of standard compounds (calycosin and formononetin) (**B**) HPLC-DAD chromatogram of the Astragalus extract.

The chemical structures of the five selected phytoestrogens are shown in Figure 4. Calibration curves showed excellent linearity (R^2^ > 0.999), ensuring sensitivity and reproducibility (Figure 5). Schizandrin (5.61 mg/g), gomisin A (1.52 mg/g), and gomisin N (2.44 mg/g) were detected in the Schisandra extract by HPLC analysis. Calycosin (0.181 mg/g) and formononetin (0.068 mg/g) were detected in the Astragalus extract. The peaks of each component were consistent with the standards (Figure 6 and Figure 7). The retention times (RTs) of the standards used for Schisandra analysis were 18.57 min for schizandrin, 19.38 min for gomisin A, and 28.25 min for gomisin N. The corresponding peaks in the Schisandra extract appeared at 18.56 min, 19.37 min, and 27.97 min, respectively. For Astragalus analysis, the RTs of the calycosin and formononetin standards were 21.89 min and 27.13 min, respectively, while the RTs observed in the Astragalus extract were 21.86 min and 27.13 min, indicating a high level of agreement. The quantification results are summarized in Table 1.

### 2.4. Analysis of vtg mRNA Expression in Hepatocytes After Phytoestrogen Treatment

**Figure 8:**
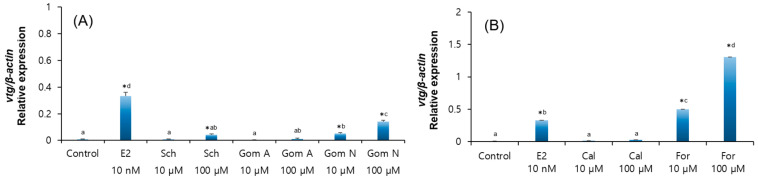
Relative expression levels of *vtg* mRNA after the treatment of eel hepatocytes with phytoestrogens. (**A**) Effects of E2 (10 nM), schizandrin (Sch), and gomisin A/N (Gom A and Gom N) at concentrations of 10 µM and 100 µM. (**B**) Effects of calycosin (Cal) and formononetin (For) at concentrations of 10 µM and 100 µM, respectively. * indicates significantly higher than the control (*p* < 0.05). Different alphabetical superscripts indicate a significant difference between treatments (*p* < 0.05).

Treatment with the standard compounds schizandrin (100 µM) and gomisin N (10 µM and 100 µM) significantly increased *vtg* mRNA expression in eel hepatocytes (Figure 8A, *p* < 0.05). Formononetin also significantly increased *vtg* mRNA expression at all concentrations, which induced higher expression than the positive control (E2) (Figure 8B, *p* < 0.05). However, calycosin and gomisin A did not show any significant differences in *vtg* mRNA expression.

## 3. Discussion

The estrogen-like activity of various plant extracts was compared to evaluate the potential utilization of phytoestrogens as an alternative to E2 during artificial maturation induction. The results showed that most of the extracts induced *vtg* mRNA expression at almost all concentrations. Among them, Schisandra and Astragalus exhibited the strongest estrogen-like activity in eel hepatocytes.

Different plant extracts had different effects on *vtg* mRNA expression, suggesting that the estrogenicity of phytoestrogen may depend on biological characteristics such as fish species or sex. When hepatocytes from sturgeon and rainbow trout were treated with the same phytoestrogens, the degree of VTG induction varied between species, and even within the same species, the response varied depending on the type of phytoestrogen [31], and in carp, differences in *vtg* expression were reported between female and male hepatocytes in response to the same concentration of E2 [32].

Schisandra extract significantly increased the expression of *esr1* and *esr2* as well as *vtg*, suggesting a possible action through a receptor-mediated pathway. However, further studies are needed to confirm whether the observed effects are indeed mediated through these estrogen receptors and to elucidate the precise mechanisms involved. In contrast, Astragalus extract induced *vtg* expression but did not show a clear association with ER expression. Although soybean extract showed relatively low *vtg* mRNA expression compared to other plant extracts, it significantly increased *esr1* mRNA expression at a concentration of 10^−3^ (*v*/*v*), exhibiting the highest level among all tested conditions. This result suggests that an increase in *esr1* expression does not always lead to a corresponding increase in *vtg* expression. This may be due to both differences in receptor binding affinity and differences in mechanisms of action, which are determined by the structural features of phytoestrogens. In a previous study, the administration of genistein and daidzein to the rainbow trout peritoneal cavity increased *vtg* expression but did not induce *esr2* and only significantly increased *esr1* [33]. Latonnelle et al. (2002) also reported differences in receptor binding affinity among phytoestrogens [34].

In this study, Schisandra extract also resulted in relatively higher cell viability than the control at low to moderate concentrations (10^−5^ to 10^−3^ (*v*/*v*)), suggesting a potential proliferative effect on hepatocytes. This may indicate that, beyond its estrogenic action, Schisandra may promote hepatocyte survival or proliferation, consistent with a previous report [35] on its antioxidant and hepatoprotective properties. However, as proliferation-specific markers were not assessed in this study, this observation should be further validated in future experiments.

In this study, HPLC analysis was performed to confirm whether the increased expression of *vtg* mRNA was indeed a response to phytoestrogens contained in the extracts. Schizandrin, gomisin A, and gomisin N were detected in Schisandra extract, with schizandrin having the highest content. Calycosin and formononetin were identified in Astragalus extract, with a higher content of calycosin. These results showed similar trends to previous studies [36,37,38], but there were differences in the content of each phytoestrogen [18]. This suggests that even in plants with the same content of phytoestrogens, the content of major components may vary depending on the extraction condition such as solvent, time, temperature, region, etc.

In addition to the main active components, several minor peaks were also observed in the HPLC chromatograms of both Schisandra and Astragalus extracts. In the Schisandra extract, multiple unidentified peaks appeared in the retention time range of 20–27 min alongside gomisin A, indicating the presence of various lignan-related compounds. These minor constituents, although not individually identified in this study, may contribute to or modulate the overall estrogen-like activity through synergistic or antagonistic interactions. Similarly, in the Astragalus extract, several peaks were observed in the same retention range beyond calycosin and formononetin, suggesting the possible presence of other bioactive isoflavones. Further studies are needed to identify and quantify these minor compounds and assess their biological relevance.

Eel hepatocytes treated with a standard compound of phytoestrogens expressed *vtg* mRNA comparable to hepatocytes treated with Schisandra and Astragalus extracts. The major compounds, formononetin and gomisin N, significantly increased *vtg* mRNA expression in a dose-dependent manner, suggesting their potential action through direct activation of estrogen receptors. In particular, formononetin showed higher *vtg* expression than the positive control E2. This result suggests that these components are the main substances that induce direct estrogen-like activity in the plant extracts. In contrast, other components present in the same extracts, namely gomisin A in Schisandra and calycosin in Astragalus, did not induce a significant increase in *vtg* expression. This highlights that individual phytoestrogens can exhibit distinct biological activities depending on their structural features and receptor binding affinities. Moreover, the estrogenic effects observed from whole plant extracts did not always match the activity of the isolated compounds. This discrepancy may be attributed to the presence of minor constituents observed in the HPLC chromatograms. It is therefore plausible that complex interactions among multiple phytochemicals in the extracts influenced the overall gene expression responses.

This study suggests that Schisandra and Astragalus extracts and their main active components, gomisin N and formononetin, can be considered as potential candidates for estrogen replacement for eel reproduction. The present study was, however, conducted only in vitro, and the mechanism of hormonal action in vivo is far more complex. Further research on the in vivo applicability of Schisandra and Astragalus extracts and their active components should be conducted prior to their use for eel maturation studies. In addition, since this study evaluated gene expression only at the mRNA level, further validation at the protein level using Western blotting is necessary to more precisely confirm the estrogenic effects.

## 4. Materials and Methods

### 4.1. Hepatocytes Culture

The experimental fish used in this study were farmed female eels (*Anguilla japonica*), which were anesthetized using 2-phenoxyethanol (Daejung Chemical & Metals, Siheung, Republic of Korea), and their livers were harvested. Primary culture of hepatocytes was performed according to the method by Iuchi et al. (2020) [39]. The hepatic portal vein was injected with 15 mL of Accutase (Innovative Cell Technologies, San Diego, CA, USA) to induce intrahepatic blood removal and cell dissociation. This was followed by washing with PBS (Gibco, Thermo Fisher Scientific, Waltham, MA, USA) containing penicillin–streptomycin (pen-strep, Gibco, Thermo Fisher Scientific, Waltham, MA, USA). After further treatment with 10 mL of Accutase, the livers were cut into small pieces and filtered sequentially through 100 μm and 70 μm cell strainers (SPL Life Sciences, Pocheon, Republic of Korea). The filtered cells were centrifuged and washed three times with the L-15 medium (Gibco, Thermo Fisher Scientific, Waltham, MA, USA) supplemented with 10% FBS (Gibco, Thermo Fisher Scientific, Waltham, MA, USA) and 1% pen-strep. Finally, the cells were seeded on poly-L-lysine (Sigma-Aldrich, St. Louis, MO, USA) coated plates and incubated at 27 °C for 4 days.

### 4.2. Ultrasonic Extraction and Filtration of Plants

Each of the ten plant sources (Schisandra fruit (Garunara, Seoul, Republic of Korea, origin: Republic of Korea), Astragalus (Garunara, Seoul, Republic of Korea, origin: Republic of Korea), guava leaf (GNBiotech, Paju, Republic of Korea, origin: Indonesia), alfalfa (Cheong-A Farm, Jeongeup, Republic of Korea, origin: USA), onion peel (Nogu, Wonju, Republic of Korea, origin: Republic of Korea), turmeric (Garunara, Seoul, Republic of Korea, origin: India), garlic (Garunara, Seoul, Republic of Korea, origin: Republic of Korea), kudzu root (Garunara, Seoul, Republic of Korea, origin: Republic of Korea), soybean (Garunara, Seoul, Republic of Korea, origin: Republic of Korea), and flaxseed (Thejoeun, Seoul, Republic of Korea, origin: Ukraine) was individually mixed with 50% (*v*/*v*) ethanol (Merck, Darmstadt, Germany) in distilled water at a 1:10 (*w*/*v*) ratio. Extraction was performed using an ultrasonic extractor (Hwashin Instrument, Seoul, Republic of Korea, PowerSonic 620) at 30 °C and 580–700 (W) for 30 min. The extract was centrifuged at 4 °C and 3000 rpm for 10 min, and the supernatant was filtered using a 0.2 μm PES Syringe Filter (Hyundai Micro, Seoul, Republic of Korea). The supernatant was considered as the stock solution (undiluted), and serial 10-fold dilutions were performed to prepare final concentrations ranging from 10^−5^ to 10^−2^ (*v*/*v*) for treatment in hepatocyte cultures. Ultrasound-assisted extraction (UAE) was selected for its simplicity, reproducibility, and high extraction efficiency [40,41]. Ethanol is one of the most commonly used solvents for phytochemical extraction [42].

### 4.3. Hepatocyte Viability Test of Plant Extracts

Cell viability was measured using the CCK-8 assay, a widely used method for assessing metabolic activity and cell viability in cytotoxicity studies due to its sensitivity and ease of use [43]. Hepatocytes were seeded at 1 × 10^5^ cells/mL in 96-well plates and stabilized for 4 days at 27 °C before treatment with plant extracts at concentrations ranging from 10^−5^ to 10^−2^. After 24 h of incubation, the medium was replaced with fresh medium containing the CCK-8 solution (Dojindo Laboratories, Kumamoto, Japan) and incubated for an additional 4 h. The absorbance at 450 nm was measured using a microplate reader (Tecan Group Ltd., Männedorf, Switzerland). Cell viability was calculated based on the following formula:

(1) $$\mathrm{Cell}\;\mathrm{viability}\left(\%\right)=\frac{B-C}{A-C}\times 100$$

where A is the absorbance of the control, B is the absorbance of the treatment group, and C is the absorbance of the blank.

### 4.4. Treatment of Hepatocytes with Plant Extracts

To avoid cytotoxic effects, concentrations (10^−5^ to 10^−3^) that did not affect cell viability were selected for treatment. Hepatocytes cultured in 24-well plates were seeded at 1.5 × 10^6^ cells/mL, treated with each plant extract, and incubated for 24 h at 27 °C. The control group was treated with the culture medium only, and the positive control was treated with 10 nM E2 (Sigma-Aldrich, St. Louis, MO, USA, E8875) under the same conditions.

### 4.5. RNA Extraction and cDNA Synthesis

After 24 h of treatment, the culture medium was removed, and total RNA was extracted from hepatocytes using 400 μL of the TRIzol™ Reagent (Ambion, Thermo Fisher Scientific, Waltham, MA, USA) according to the manufacturer’s protocol. Chloroform (Sigma-Aldrich, St. Louis, MO, USA) was added 80 μL, and the samples were vortexed and incubated at room temperature for 2 min, followed by centrifugation at 12,000× *g* for 20 min at 4 °C. The supernatant was transferred to a new tube, mixed with 200 μL of isopropanol (Sigma-Aldrich, St. Louis, MO, USA) and incubated for 10 min at room temperature. After centrifugation (12,000× *g*, 10 min, 4 °C), the RNA pellet was washed with 75% ethanol, centrifuged again (7600× *g*, 5 min, 4 °C), and air-dried. RNA concentration and purity were determined using a NanoDrop 2000 spectrophotometer (ThermoScientific, Waltham, MA, USA). Complementary DNA (cDNA) was synthesized using TOPscript™ RT DryMIX (Enzynomics, Daejeon, Republic of Korea) following the manufacturer’s instructions. TRIzol was used due to its high yield and integrity. NanoDrop was chosen for its accuracy. The cDNA kit is widely used and compatible with qPCR.

### 4.6. Quantitative Real-Time PCR

qRT-PCR was performed using Topreal™ qPCR 2X PreMIX SYBR Green (Enzynomics, Daejeon, Republic of Korea), and gene expression was analyzed by relative quantification using *β-actin* as the reference gene. The primer sequences were adopted from the study by Hyeon et al. (2019) [44] and are listed in Table 2.

### 4.7. HPLC Analysis

Qualitative and quantitative analyses of the major phytoestrogens by HPLC were performed using the undiluted supernatant of the Schisandra and Astragalus extracts, which showed the highest induction of *vtg* mRNA expression. The extracts used in the experiments were filtered through a 0.22 μm PVDF membrane filter and used as test solutions. Each standard (schizandrin (≥98% purity, Sigma-Aldrich, St. Louis, MO, USA, SML0054), gomisin A (≥98% purity, Wako Pure Chemical Industries, Osaka, Japan, 077-04951), gomisin N (≥98% purity, Wako Pure Chemical Industries, Osaka, Japan, 074-04961), calycosin (≥98% purity, Sigma-Aldrich, St. Louis, MO, USA, B9938), formononetin (≥98% purity, Sigma-Aldrich, St. Louis, MO, USA, 94334) was dissolved in methanol (Sigma-Aldrich, St. Louis, MO, USA), appropriately diluted, and used for calibration. These standards were selected based on previous studies identifying them as major phytoestrogens present in Schisandra and Astragalus [28,29,30].

HPLC analysis was performed using an Agilent 1260 Infinity II Quat Pump system (Agilent Technologies, Santa Clara, CA, USA) equipped with a YMC-Pack Pro C18 column (4.6 × 250 mm, 5 μm, YMC, Kyoto, Japan). The mobile phase consisted of solvent A (water with 0.1% TFA) and solvent B (acetonitrile with 0.1% TFA). Samples (10 μL) were injected at a flow rate of 1.0 mL/min, and detection was performed at 254 nm using a DAD WR detector. For the Schisandra extract, the gradient conditions were as follows: 0–15 min, 80:20 (% *v*/*v*, A:B); 15–25 min, 30:70; 25–35 min, 18:82; 35–40 min, 100% B; and 40–55 min, returned to 80:20. For the Astragalus extract, the conditions were: 0–15 min, 85:15 (% *v*/*v*, A:B); 15–20 min, 68:32; 20–27 min, 60:40; 27–30 min, 35:65; 30–38 min, 100% B; 38–52 min, returned to 85:15; and then gradually adjusted to 80:20.

Quantification was carried out based on calibration curves constructed using standard solutions (R^2^ > 0.999), and the contents were calculated using the following formula:

(2) $$\mathrm{Content}\left(\frac{\mathrm{mg}}{g}\right)=\frac{C\times V\times D\times P}{W}$$

where C is the concentration of the standard compound in the test solution (mg/mL), V is the total volume (mL), D is the dilution factor, P is the purity of the standard compound, and W is the weight of the sample (g).

### 4.8. Treatment of Hepatocytes with Phytoestrogens

Hepatocytes from female eels were isolated and cultured according to established methods. After stabilization for 4 days, the cells were treated with standard compounds (schizandrin, gomisin A, gomisin N, calycosin, and formononetin) identified by HPLC analysis at concentrations of 1 µM and 100 µM. Each compound was dissolved in methanol and diluted to the final concentration. E2 (10 nM) was used as a positive control. Total RNA was extracted 24 h after treatment, and cDNA was synthesized. The expression level of *vtg* mRNA was analyzed by qRT-PCR.

### 4.9. Statistical Analysis

All experimental results are expressed as the mean ± the standard error of the mean (SEM). Differences in CCK-8 assay results and *esr* and *vtg* mRNA expression between the control and each treatment were analyzed using independent samples t-tests. For *vtg* mRNA expression, which showed significant differences in most treatments compared to the control, one-way ANOVA was performed, followed by Duncan’s post hoc test (SPSS Statistics 20, *p* < 0.05).

## Figures and Tables

**Table 1:** Quantification of major phytoestrogens in Schisandra and Astragalus extracts by HPLC.

Compound	Plant Source	RT (Standard, min)	RT (Sample, min)	Content (mg/g Extract)
Schizandrin	Schisandra	18.57	18.56	5.61
Gomisin A	Schisandra	19.38	19.37	1.52
Gomisin N	Schisandra	28.25	27.97	2.44
Calycosin	Astragalus	21.89	21.86	0.181
Formononetin	Astragalus	27.13	27.13	0.068

**Table 2:** Primer sequence for quantitative real-time PCR.

Gene		Primer Sequence	Product Size
*β-actin*	F	GAGACCTTCAACACCCCAG	95
	R	CCAGAGTCCATCACAATACCAG	
*vtg*	F	GACAGTGTAGTGCAGATGAAG	117
	R	ATAGAGAGACAGCCCATCAC	
*esr1*	F	TTTCGACATGATCCTCGCCA	102
	R	GCACCGGAGTTGAGCAGTAT	
*esr2* [44]	F	AAGTACACCTGCTGGAGTGC	220
	R	AGGCACACATACTCCTCCCT	

## Data Availability

The data from this study can be found in the article.

## Abbreviations

The following abbreviations are used in this manuscript:

E2	17β-estradiol
esr	Estrogen receptor
vtg	Vitellogenin
HPLC	High-performance liquid chromatography
Sch	Schizandrin
Gom	Gomisin
Cal	Calycosin
For	Formononetin
